# RECCAS - REmoval of Cytokines during CArdiac Surgery: study protocol for a randomised controlled trial

**DOI:** 10.1186/s13063-016-1265-9

**Published:** 2016-03-12

**Authors:** Andreas Baumann, Dirk Buchwald, Thorsten Annecke, Martin Hellmich, Peter K Zahn, Andreas Hohn

**Affiliations:** Department of Anaesthesiology, Intensive Care, Palliative Care and Pain Medicine, BG University Hospital Bergmannsheil, Ruhr-University Bochum, Buerkle-de-la-Camp-Platz 1, Bochum, 44789 Germany; Department of Cardiac and Thoracic Surgery, BG University Hospital Bergmannsheil, Ruhr-University Bochum, Buerkle-de-la-Camp-Platz 1, 44789 Bochum, Germany; Department of Anaesthesiology and Intensive Care Medicine, University Hospital of Cologne, Kerpener Str. 62, 50937 Cologne, Germany; Institute of Medical Statistics, Informatics and Epidemiology, University of Cologne, Kerpener Straße 62, 50924 Cologne, Germany

**Keywords:** Haemoadsorption, Cardiac surgery, Cardiopulmonary bypass, Cytokines, Inflammation, Glycocalyx shedding

## Abstract

**Background:**

On-pump cardiac surgery triggers a significant postoperative systemic inflammatory response, sometimes resulting in multiple-organ dysfunction associated with poor clinical outcome. Extracorporeal cytokine elimination with a novel haemoadsorption (HA) device (CytoSorb®) promises to attenuate inflammatory response. This study primarily assesses the efficacy of intraoperative HA during cardiopulmonary bypass (CPB) to reduce the proinflammatory cytokine burden during and after on-pump cardiac surgery, and secondarily, we aim to evaluate effects on postoperative organ dysfunction and outcomes in patients at high risk.

**Methods/design:**

This will be a single-centre randomised, two-arm, patient-blinded trial of intraoperative HA in patients undergoing on-pump cardiac surgery. Subjects will be allocated to receive either CPB with intraoperative HA or standard CPB without HA. The primary outcome is the difference in mean interleukin 6 (IL-6) serum levels between the two study groups on admission to the intensive care unit. A total number of 40 subjects was calculated as necessary to detect a clinically relevant 30 % reduction in postoperative IL-6 levels. Secondary objectives evaluate effects of HA on markers of inflammation up to 48 hours postoperatively, damage to the endothelial glycocalyx and effects on clinical scores and parameters of postoperative organ dysfunction and outcomes.

**Discussion:**

In this pilot trial we try to assess whether intraoperative HA with CytoSorb® can relevantly reduce postoperative IL-6 levels in patients undergoing on-pump cardiac surgery. Differences in secondary outcome variables between the study groups may give rise to further studies and may lead to a better understanding of the mechanisms of haemoadsorption.

**Trial registration:**

German Clinical Trials Register number DRKS00007928 (Date of registration 3 Aug 2015)

## Background

Cardiac surgery, particularly on-pump, causes a systemic inflammatory response syndrome (SIRS) with a marked release of cytokines. Proinflammatory mediators, such as IL-6, IL-8, and TNF-alpha reach peak levels 2 to 4 hours after termination of cardiopulmonary bypass (CPB) and decrease to almost normal levels within 24 hours [1]. SIRS after cardiac surgery is associated with postoperative multiorgan system dysfunction and major complications [2, 3]. The proinflammatory cytokine burden correlates with poor postoperative outcome [4]. So far, intraoperative use of leucocyte filtration, endotoxin adsorption, or ultrafiltration during CPB in cardiac surgery has not been able to show consistent results in removal of cytokines or other inflammatory mediators. Although use of adsorption and filtration devices was safe and well-tolerated, studies failed to demonstrate positive effects on clinical outcome [5–8].

A novel extracorporeal sorbent haemoadsorption (HA) device (CytoSorb®) was recently developed for cytokine removal from the blood and is now approved in the European Union. It is broadly indicated for any clinical situation where cytokine levels are elevated. CytoSorb® is clinically proven to reduce cytokine storm in a multi-centre, randomised European Sepsis Trial conducted in Germany. Haemoadsorption was safe and well-tolerated in more than 300 human treatments in critically ill patients with sepsis and lung injury [9], and has been safe in more than 650 human treatments overall. Early data suggests that HA with CytoSorb® can reduce organ injury and improve survival in patients at high risk, particularly those with very high cytokine levels and who are older than age 65. A recent systematic review and meta-analysis [10] concluded that among current adsorbing techniques, CytoSorb® may be the most promising due to data from animal studies [11–13] and first clinical results [14].

To date there is limited data that HA with CytoSorb® is able to reduce the effects of the inflammatory response after cardiac surgery. However, positive clinical effects at our institution have led to a broader use of this novel technique in patients at high risk for postoperative complications. In this pilot trial we investigate whether an intraoperative use of HA with CytoSorb® in the CPB circuit can effectively reduce postoperative levels of proinflammatory cytokines and may attenuate postoperative systemic inflammatory response.

## Methods/design

**Fig. 1:**
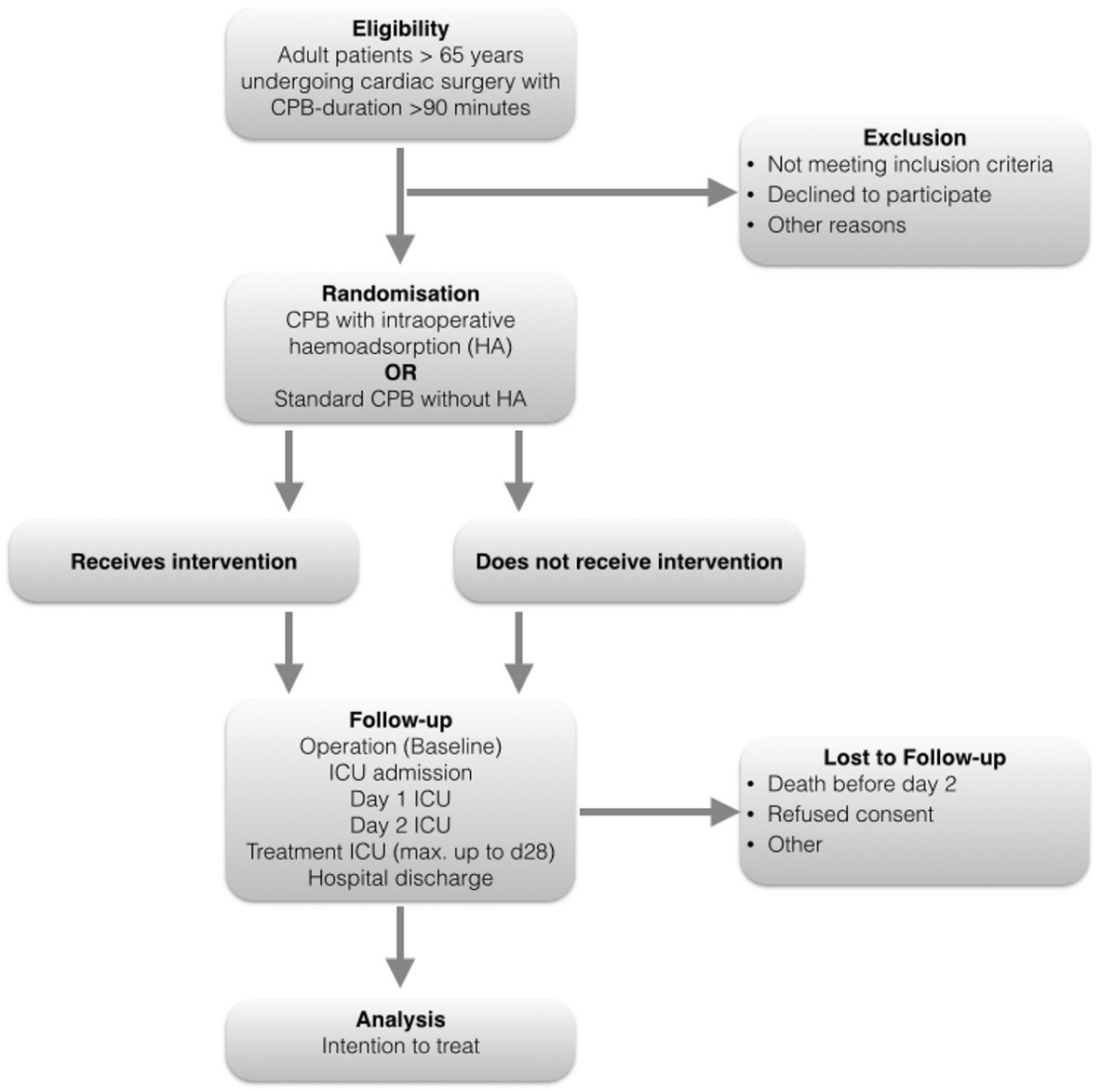
Consolidated Standards of Reporting Trials (CONSORT) flow diagram of the REmoval of Cytokines during CArdiac Surgery (RECCAS) trial. A single-centre randomised, two-arm, controlled, patient-blinded trial of the effects of intraoperative haemoadsorption on postoperative inflammatory response and organ dysfunction in patients undergoing on-pump cardiac surgery

This will be a single-centre randomised, two-arm, patient-blinded trial of the effects of intraoperative HA on postoperative inflammatory response and organ dysfunction in patients undergoing on-pump cardiac surgery. Trial flow per the Consolidated Standards of Reporting Trials (CONSORT) guidelines [15] is shown in Fig. 1.

### Ethics

The study was approved by the Ethics committee of the Medical Faculty of the Ruhr University Bochum (reference number 5094–14) and the study was registered in the German Clinical Trials Register (registry number DRKS00007928). The study will be conducted in accordance with the Helsinki Declaration and the ICH-GCP guidelines. Written informed consent will be obtained from all subjects prior to study inclusion and randomisation.

### Study intervention

**Fig. 2:**
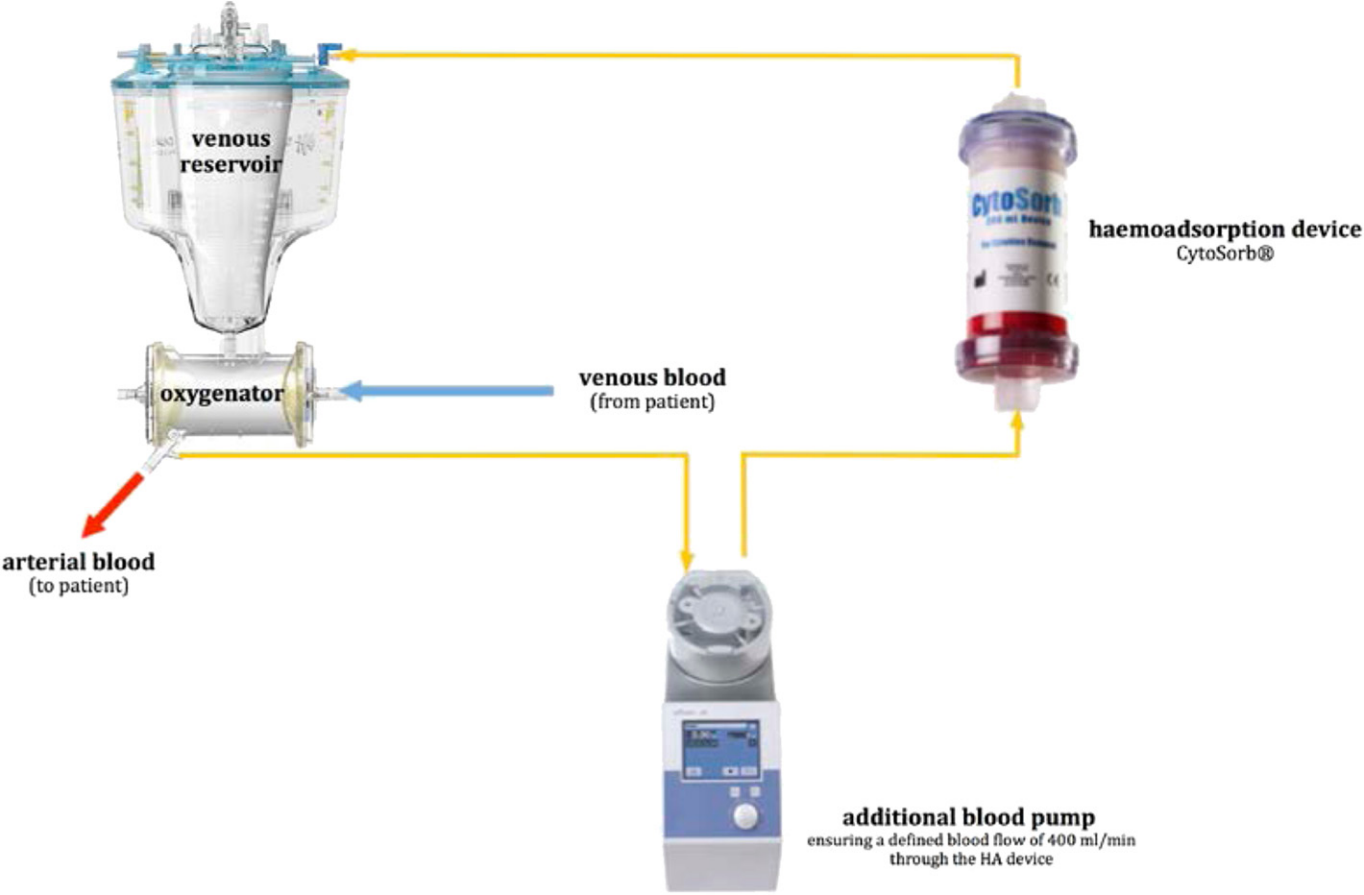
Integration of the haemoadsorption device into the cardiopulmonary bypass (CPB) circuit. Oxygenated blood is taken from the oxygenator of the heart-lung machine (HLM), and an additional blood pump ensures a defined blood flow of 400 mL/min through the adsorber to the venous reservoir of the HLM

Subjects will be randomly allocated to receive either intraoperative HA during cardiopulmonary bypass (CPB) or standard CPB without HA. The HA device will be included in the CPB circuit between the oxygenator and the venous reservoir. An additional pump will ensure a defined blood flow of 400 mL/min through the HA device (Fig. 2). The study will be conducted at the BG University Hospital Bergmannsheil (Bochum, Germany).

### Recruitment and data collection

Subjects for the study will be enrolled only at predefined study days. Study days depend on the availability of a perfusionist trained on the use of CytoSorb®, and a heart-lung machine (HLM) approved for the inclusion of the haemoadsorber in the CPB circuit. Furthermore, at least one member of the study team has to be on service on the day of surgery as well as on ICU days 1 and 2. On the day before a predefined study day, the OR plan will be screened for patients eligible for the study. The OR plan is made by a team not associated to the study group. Eligible patients will be asked to consent to the study. A list of patients screened for inclusion criteria at predefined study days will be generated, and data on the recruitment process will be published with the final manuscript.

Clinical data and data on outcome parameters will be documented in a paper case report form (CRF) during daily visits by the members of the study team. Afterwards, all data will be transferred to REDCap™ (Research Electronic Data Capture), a web-based database for research studies. REDCap™ is a secure, web-based application designed to support data capture for research studies, providing 1) an intuitive interface for validated data entry; 2) audit trails for tracking data manipulation and export procedures; 3) automated export procedures for seamless data downloads to common statistical packages; and 4) procedures for importing data from external sources [16]. Paper CRFs and the electronic database will be stored for at least 10 years according to the ICH-GCP guidelines.

### Randomisation and blinding

Assignment to the two treatment arms will be performed on a 1:1 basis using a computer-generated system after induction of anaesthesia before the beginning of surgery. Patients will be kept blinded. The lab analyst will also be blinded to patients’ data and to the different study groups. Thus, laboratory outcome parameters are analysed independently from the clinical data.

### The haemoadsorption device (CytoSorb®)

The CytoSorb® adsorber is composed of biocompatible porous polymer beads able to remove substances from whole blood based on pore capture and surface adsorption. Substances that are larger than the pores, such as blood cells, cannot get into the pores and go around the beads. Very small substances, such as electrolytes and other blood chemistry components, are also not captured. Appropriately sized molecules in the 5–60 kDa range, however, become trapped in the vast network of pores and channels in every bead, and are permanently eliminated from the blood. CytoSorb® was specifically designed to target this molecular weight range, given that the majority of cytokines and inflammatory mediators fall within this size spectrum. The technology is not an affinity-based sorbent and does not use antibodies, ligands, cells or drugs [17].

Table 1 shows technical data of the CytoSorb® adsorber according to the manufacturer information [17]. CytoSorb® is CE Mark approved under the Medical Devices Directive, is ISO 10993 biocompatible, and is manufactured in the United States under ISO 13485 certification. Over 5,500 treatments have been performed in more than 200 centres around the world. The device is considered safe and well-tolerated. CytoSorb® has standard dialysis connectors that allow integration with haemodialysis machines and heart-lung machines found in most hospitals today. It is compatible with either systemic heparin or regional citrate anti-coagulation, with the same anti-coagulation requirements as for dialysis or on-pump cardiac surgery [17]. Based on the Company’s European Sepsis Trial [9], there is no adverse removal of leukocytes or red blood cell haemolysis during treatment. As with most extracorporeal therapies, some platelets are removed during therapy, but the amount was less than 10 % per treatment and was not clinically significant. Average albumin removal was less than 10 % after seven days of CytoSorb® therapy and not clinically significant [9].

**Table 1:** Technical data of the CytoSorb® adsorber according to the manufacturer information

Extracorporeal blood volume:	120 mL
Blood flow rates min-max	100–400 mL/min
Max. treatment duration	24 hours
Anticoagulation	Possible with heparin or citrate
Sterilisation	Gamma sterilisation
Further details	Latex- and polyhexahydrotriazines-free product
Storage conditions	1 to 40 °C; upright storage

Modified from [16]

### Inclusion criteria

- Adult patients > 65 years undergoing cardiac surgery with an expected CPB duration >90 minutes
- Written informed consent

### Exclusion criteria

- Age < 65 years
- Missing informed consent
- Planned CPB temperature < 32 °C
- Emergency surgery
- Acute infective endocarditis
- AIDS with a CD4 count of < 200/μL
- Previous renal replacement therapy
- Pre-existing kidney disease not requiring RRT (GFR < 30 mL/min)
- Prior kidney transplant
- Application of contrast medium on the day of surgery
- Immunosuppressive therapy or long-term therapy with corticosteroids
- Participation in another clinical intervention trial

### Standardisation of anaesthesia, CPB management and ICU treatment

All patients will receive standard anaesthetic treatment. Intraoperative transoesophageal echocardiography is performed for optimisation of haemodynamic management if indicated.

Standard of care treatment in the ICU is given to all study subjects. Especially for mechanical ventilation, nutrition, sedation, anticoagulation, and blood glucose control, therapy is based on local treatment protocols.

In the ICU, treatment with vasopressors, inotropes, and fluids is guided by haemodynamic monitoring using a transpulmonary thermodilution technique (PiCCO®) and derived dynamic parameters. Echocardiography (transthoracic or transoesophageal) is performed daily in ICU patients and whenever indicated. Optimisation of haemodynamics and preload guided by PiCCO® and/or echocardiography is mandatory before starting renal replacement therapy (RRT).

RRT will be started in the presence of absolute indications. Absolute indications according to KDIGO (Kidney Disease: Improving Global Outcomes) guidelines are when life-threatening refractory changes in fluid, electrolyte, and acid–base balance (for example, hyperkalaemia, acidaemia, pulmonary oedema, uraemic complications) exist [18]. RRT will also be initiated in patients with acute kidney injury (AKI) with urine output <0.3 mL/kg/h for ≥24 hours or anuria for ≥12 hours (AKIN 3). In patients with AKIN 2, RRT should be started when concomitant ongoing organ failure (development or progression of non-renal Sequential Organ Failure Assessment [SOFA] organ system subscore ≥2) and/or haemodynamic instability (norepinephrine/epinephrine ≥0.1 μg/kg/min or use of terlipressin) are present. Continuous veno-venous haemodialysis (CVVHD) (30 mL/kg/h) with regional citrate-anticoagulation is used in all ICU patients with AKI. RRT will be discontinued if renal recovery defined by urine output (>400 mL/24 h) and creatinine clearance (>20 mL/min) occurs [19].

### Primary objective

Table 2 shows an overview on the time points of routine blood samples and outcome laboratory parameters during the study period. The study aims to assess the efficacy of intra-operative haemoadsorption with CytoSorb® to remove cytokines from circulation for prevention of surgical associated inflammatory response and complications in patients at high risk.

**Table 2:** Summary of laboratory parameters at different time points

			During CPB^a^							
	Hospital admission	Baseline	10 min	30 min	60 min	ICU admission	ICU d 1	ICU d 2	ICU d 3-28	Follow-up (hospital discharge)
Routine blood samples	X	X				X	X	X	X	X
IL-6		X	X	X	X	X	X	X		
IL-2		X	X	X	X	X	X	X		
IL-8		X	X	X	X	X	X	X		
IL-10		X	X	X	X	X	X	X		
TNF-alpha		X	X	X	X	X	X	X		
C3a		X	X	X	X	X	X	X		
Free haemoglobin		X	X	X	X	X	X	X		
Haptoglobin		X	X	X	X	X	X	X		
Myoglobin		X	X	X	X	X	X	X		
Fibrinogen		X	X	X	X	X	X	X		
Syndecan-1		X	X	X	X	X	X	X		
Hyaluronan		X	X	X	X	X	X	X		
Heparan sulphate		X	X	X	X	X	X	X		
Markers of mast cell degranulation		X	X	X	X	X	X	X		

^a^Intraoperative cytokine kinetics and markers of secondary objectives will only be assessed in patients allocated to the haemoadsorption device at 10, 30 and 60 min after initiation of cardiopulmonary bypass (CPB). For this, blood samples at the defined time points will be taken from the CPB circuit both before and after use of the haemoadsorption device

- The primary outcome is the difference in mean IL-6 serum levels between the two study groups on admission to the ICU.

### Secondary objectives

- Length of hospital stay
- Length of ICU stay
- ICU mortality
- Hospital mortality
- Surveillance of vital parameters in the ICU (haemodynamic and ventilation parameters and data for support of other organs in the ICU will be monitored due to the standard of care)
- Daily SOFA organ failure scores in the ICU and on hospital discharge
- Incidence of postoperative delirium (NuDESC scores)
- Infections
- Incidence of acute kidney injury (AKI) of any stage according to the AKIN classification [18]
- Duration of renal support
- Renal function on hospital discharge
- Serum markers of glycocalyx shedding (syndecan-1, heparan-sulphate and hyaluronan)
- Blood kinetics of free haemoglobin, myoglobin and haptoglobin
- Hepatic function (liver enzymes, indocyanine green [ICG] plasma disappearance rate [PDR])
- Markers of mast cell degranulation (for example, mast cell tryptase)
- Blood and urine cytokine kinetics

Cytokine levels (such as IL-10 and TNF-alpha) and other routinely used biomarkers (such as CRP and PCT) will be assessed daily from before start of CPB up to 48 hours after ICU admission. In the HA group, additionally blood samples for primary and secondary laboratory outcome parameters are taken from the CPB circuit before entering and after the passage of the HA device at 10, 30 and 60 minutes after CPB start (Table 2).

### Clinical data collected during the study period

Baseline data:

- Demographic data
- Secondary diagnoses
- Medication
- Laboratory parameters
- Clinical scores (ASA, Euroscore, Thakar score, NuDESC)

Anaesthesia and surgery:

- Duration of operation, CPB and aortic cross-clamping
- Vasopressors and inotropics
- Fluids and fluid balance
- Transfusion and coagulation factors
- Urine output
- Baseline IL-6 concentrations (after induction of anaesthesia) in all patients. Intraoperative IL-6 kinetics in patients with haemoadsorption.
- Baseline concentration of secondary laboratory outcomes (after induction of anaesthesia) in all participants. Secondary laboratory outcomes (for example, cytokines, free haemoglobin, myoglobin) at 10, 30 and 60 minutes after CPB start, only in patients with haemoadsorption.

ICU:

- Daily SOFA scores
- Haemodynamics (including cardiac output monitoring)
- Mechanical ventilation
- Postoperative delirium
- Infectious complications, antibiotics
- Fluid balance
- Renal function, renal replacement therapy
- Cytokine concentrations (including IL-6) up to day 2 in the ICU
- Secondary laboratory outcomes up to day 2 in the ICU

Follow-up (at hospital discharge):

- Length of stay in the ICU
- Length of stay in the hospital
- Renal function on ICU/hospital discharge
- Readmission to ICU
- Cause of death

### Sample size calculation and statistical analysis

Based on previous research we expect a mean IL-6 concentration of 200 (SD 50) pg/mL in the control group [7, 20–22] and a reduction of 30 % (that is, 140 (SD 35) pg/mL) by intraoperative haemoadsorption with CytoSorb®. The Welch-modified *t*-test requires 15 patients per group to reach a power of 95 % at two-sided significance level 5 % (calculated with Stata 14.1; StataCorp, College Station, TX, USA; command “power two means 200 140, sd1(50) sd2(35) power(0.95)”). To compensate for non-evaluable patients and, possibly, the use of non-parametric methods, 20 patients per group will be included and randomised.

The full analysis set is derived from the intention-to-treat principle, meaning that all patients randomised and operated on will be analysed as assigned—apart from non-evaluable patients, that is, those without a valid IL-6 serum level measurement on ICU admission. The Welch-modified *t*-test (with corresponding 95 % confidence) interval will be applied to test the null hypothesis of equal mean IL-6 serum levels in treatment groups on ICU admission. In the case of relevantly skewed (non-normal) data distributions within groups, the non-parametric Wilcoxon rank sum test (with corresponding 95 % confidence interval) will be applied.

Secondary outcome measures in the two treatment groups (all available observations) will be summarised descriptively either by count and percentage or by mean, standard deviation and percentiles (0, 25, 50, 75, 100), contingent on distributional characteristics. Adverse event data will be listed and aggregated by category. Significance tests and confidence intervals will be calculated to support interpretation. A subgroup analysis will be performed by sex (assumed inclusion ratio 1.5 (male): 1.0 (female)). Statistical calculations will be done with SPSS Statistics software (IBM Corp., Armonk, NY, USA).

## Discussion

In this pilot trial we will assess if intraoperative haemoadsorption with CytoSorb® can significantly reduce postoperative IL-6 serum levels in patients undergoing on-pump cardiac surgery. The adsorber has been successfully used in different clinical settings, and the number of published case reports is steadily increasing [23–26]. However, little is known about the effects of HA with CytoSorb® during on-pump cardiac surgery. Thus, the present study was designed to learn about the effects of HA on intra- and postoperative patterns of systemic inflammation and of organ dysfunction. We chose the difference of serum IL-6 levels on ICU admission as the primary outcome, as IL-6 is a typical proinflammatorily acting cytokine. IL-6 levels rise significantly after cardiac surgery and peak 2 to 4 hours after termination of CPB. Elevated levels of IL-6 are associated with postoperative myocardial ischaemia, development of low cardiac output, and requirement for vasopressors [27, 28]. High levels of IL-6 on ICU admission after cardiac surgery also correlate well with the development of postoperative infections [22]. Thus, reducing IL-6 from the CPB circuit may attenuate the postoperative inflammatory response. For our study we defined a 30 % reduction of IL-6 to be clinically relevant. However, our study also focuses on other mediators of inflammation as secondary outcomes. Increased levels of IL-8 are associated with length of postoperative inotropic support and duration of mechanical ventilation, and TNF-alpha levels correlated with impaired left ventricular contractility [1]. Furthermore, kinetics of IL-10, a typical representative of an anti-inflammatory cytokine, will also be evaluated. IL-10 modulates the compensatory anti-inflammatory response syndrome (CARS) counteracting SIRS [29–31]. An overwhelming anti-inflammatory response can lead to immunosuppression and susceptibility to postoperative infections.

Our study also aims to evaluate other mechanisms of postoperative organ failure. Increased levels of free haemoglobin and myoglobin contribute to the pathogenesis of cardiac surgery-associated AKI (CSA-AKI) [32]. As CytoSorb® effectively removes cytokines with a molecular weight of approximately 10–50 kDa, one can assume that both free haemoglobin and myoglobin are removed from the CPB as well. This may have a further protective effect on the kidney and probably contributes to preservation of renal function after cardiac surgery.

Another consequence of hypoxia, ischaemia and reperfusion is damage of the endothelial glycocalyx [33, 34], probably by a purine mediated mast cell-dependent mechanism [35, 36]. In turn, degradation products (such as syndecan-1, hyaluronan and heparan-sulphate) may increase inflammatory response through toll-like receptor 4 (TLR-4)-mediated positive feedback mechanism. Thus, in this study we also try to assess whether circulating glycocalyx degradation products are eliminated by intraoperative haemoadsorption, and if inflammatory response is accompanied by decreased glycocalyx shedding.

In the haemoadsorption group, we additionally evaluate the ability of CytoSorb® to significantly adsorb different cytokines and other mediators of interest during CPB. For this, blood samples for primary and secondary laboratory outcome parameters are taken before entering and after the passage of the HA device at 10, 30 and 60 minutes after CPB start.

Finally our pilot study provides a link between insights into inflammatory response after intraoperative HA and clinical outcomes. Possible differences in clinical outcome parameters, such as mortality, length of stay, numbers of complications, infections and SOFA scores between the study groups may give rise to further studies and possibly lead to a better understanding of mechanisms of haemoadsorption. As this is a clinical study, one limitation could be that group differences in CBP duration may influence peak levels of cytokines. Furthermore, the interval between end of CPB and ICU admission is not equal in all patients. But from our clinical experience we know that those differences are negligible, and we assume that effects on cytokine levels are not of relevance.

## Trial status

This randomised pilot trial was designed as an investigator-initiated study for which BG University Hospital Bergmannsheil (Bochum, Germany) was assumed to be acting as sponsor The first participant was included on 26 January 2015. To date, 17 participants have been recruited.

## Abbreviations

AIDS: acquired immune deficiency syndrome
AKI: acute kidney injury
AKIN: Acute Kidney Injury Network
ASA: American Society of Anesthesiologists
CD4: cluster of differentiation 4
CE: CE declaration
CONSORT: Consolidated Standards of Reporting Trials
CPB: cardiopulmonary bypass
CRP: C-reactive protein
CSA-AKI: cardiac surgery-associated acute kidney injury
CVVHDF: continuous veno-venous haemodiafiltration
DO_2_I: oxygen delivery index
GDP: goal directed perfusion
HA: haemoadsorption
HLM: heart-lung machine
ICG: indocyanine green
ICH-GCP: International Conference on Harmonisation-Good Clinical Practice
ICU: intensive care unit
IL: interleukin
ISO: International Organization for Standardization
KDIGO: Kidney Disease: Improving Global Outcomes
NuDESC: Nursing Delirium Screening Scale
PCT: procalcitonin
PDR: plasma disappearance rate
RRT: renal replacement therapy
SIRS: systemic inflammatory response syndrome
SOFA: Sequential Organ Failure Assessment
TLR-4: toll-like receptor 4
TNF-alpha: tumor necrosis factor alpha
